# Supplementary material for: Species-Level Taxonomic Characterization of Uncultured Core Gut Microbiota of Plateau Pika
Source: Microbiol Spectr. 2023 Apr 17;11(3):e03495-22. doi: 10.1128/spectrum.03495-22 (PMC10269723; doi:10.1128/spectrum.03495-22)
Supplement: Supplemental file 10 — Fig. S1 to S8. Download spectrum.03495-22-s0001.pdf, PDF file, 6.8 MB [file spectrum.03495-22-s0001.pdf]

Contents

Figures

Figure S1. Error rate for 16S rRNA sequences 2

Figure S2. Assessment of the genomes' quality 2

Figure S3. Relationship between plateau pika, gut microbiota and alpine ecosystem... 3

Figure S4. Phylogenetic analysis of Core OPUs 4

Figure S5. The core microbiota of intestinal content of Plateau Pika 5

Figure S6. The AAI and ANI value of SGBs 6

Figure S7. Profile of CAZymes detected in the SGBs 7

Figure S8. Taurine desulfonation pathway in SGBs 8

Tables

Table S1. FL16S amplicon metadata

Table S2. OPU table

Table S3. Core OPU 16S rRNA identity & relative abundance

Table S4. Metagenomic samples information

Table S5. SGBs information

Table S6. SGBs abundance

Table S7. SGBs AAI&ANI

Table S8. SGBs coregenes

Table S9. CAZymes and metabolites

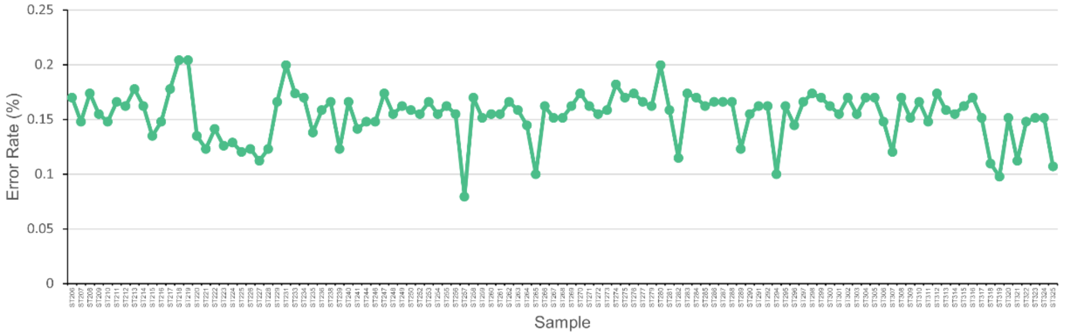

Figure S1. Error rate for 16S rRNA sequences

Each dot represents each sample's estimated full-length 16S rRNA sequencing error rate.

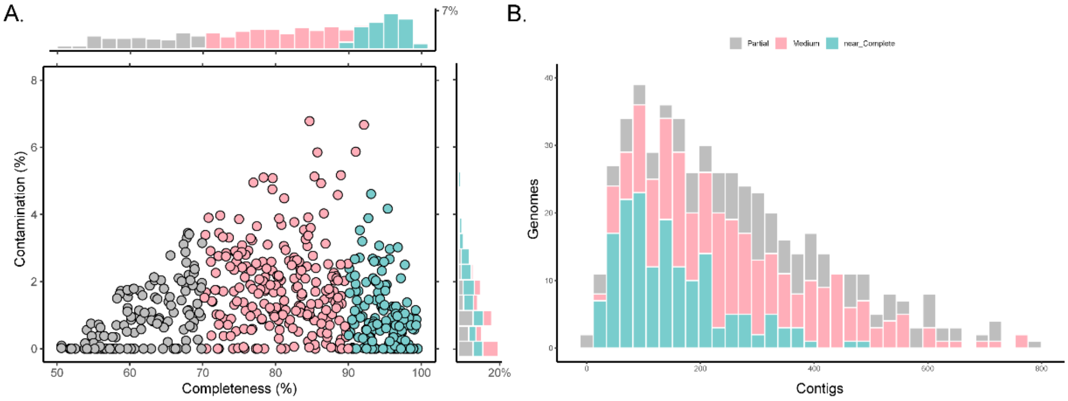

Figure S2. Assessment of the genomes' quality

Estimated completeness and contamination of genomes recovered from 16 pika metagenomes. Genome quality was defined as completeness − 5 × contamination, and only genomes with quality of ≥ 50 were retained.

(A) Near-complete genomes (completeness ≥ 90%; contamination ≤ 5%) are shown in blue, medium-quality genomes (completeness ≥ 70%; contamination ≤ 10%) in pink, and partial genomes (completeness ≥ 50%; contamination ≤ 4%) in grey. Histograms along the x and y axes show the percentage of genomes at varying levels of completeness and contamination, respectively;

(B) Number of contigs comprising each genome with colours indicating genome quality.

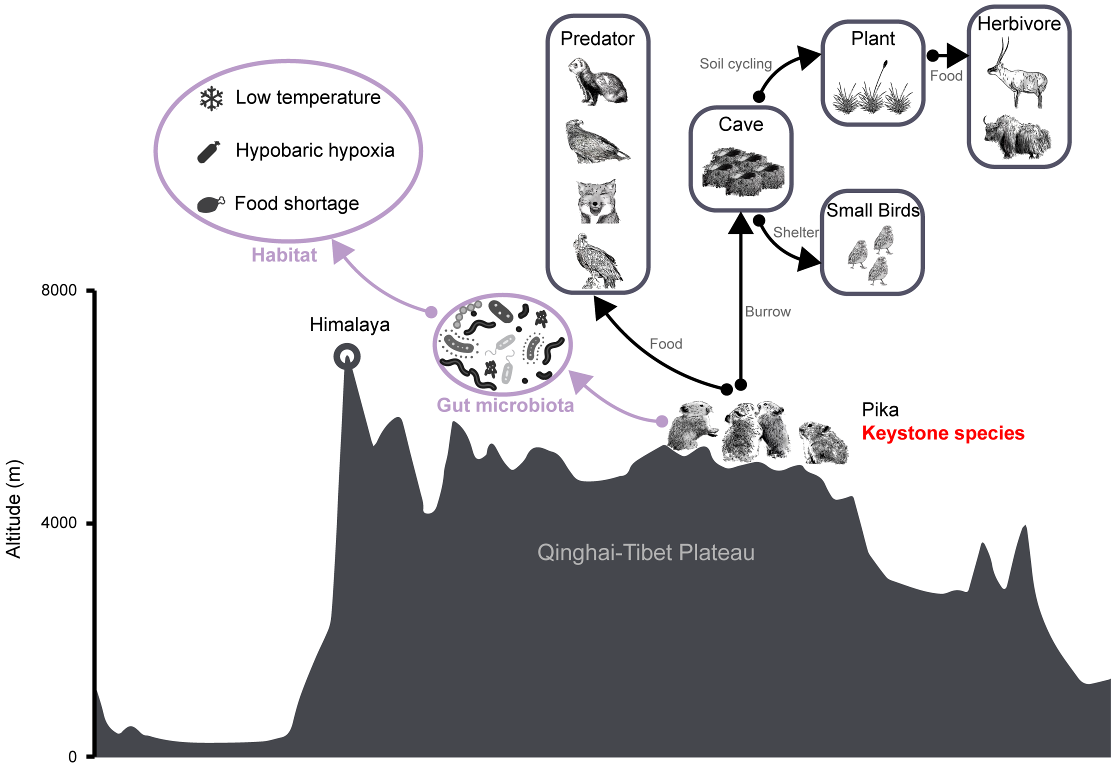

Figure S3. Relationship between plateau pika, gut microbiota and alpine ecosystem

The plateau pika (*Ochotona curzoniae*) is endemic to the alpine meadow of Qinghai-Tibetan Plateau. Plateau pika as a keystone species is critical to maintaining the stability of alpine ecosystems by nesting for burrow living birds, promoting soil substance cycling, and supplying food resources for a series of predators for energy conversion. The microbiota inhabiting the gut of plateau pika may indirectly influence the maintenance of ecological stability because of their high energy conversion efficiency and the possible involvement of their metabolites in environmental adaptation.

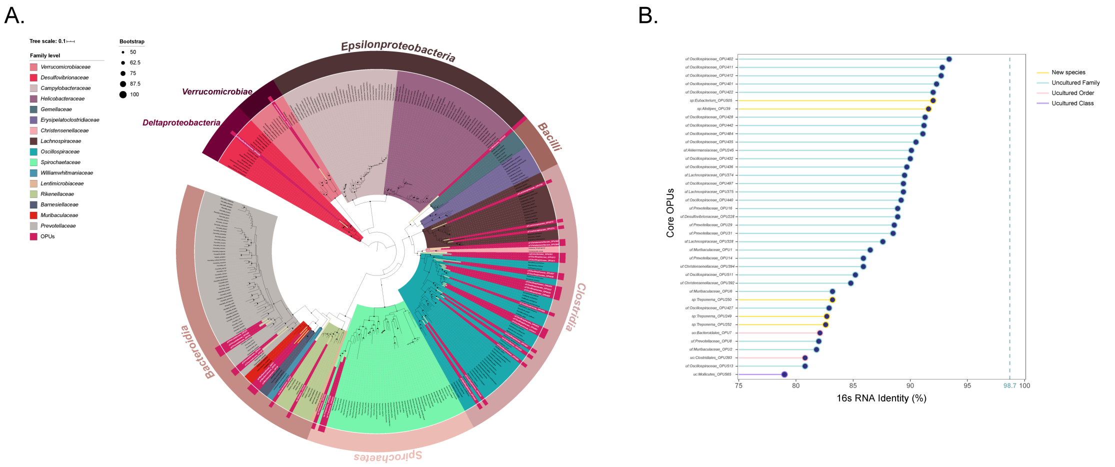

Figure S4. Phylogenetic analysis of Core OPUs

Pink labels are core OPUs, additionally incorporating affiliated ref strains' 16s rRNA sequences from LTP database.

(A) Core OPUs phylogenetic tree was produced from full-length 16s rRNA sequences using RAxML, and subsequently drawn using iTOL;

(B) The maximum identity value of core OPUs compared with affiliated ref strains were calculated using Ortho-ANI;

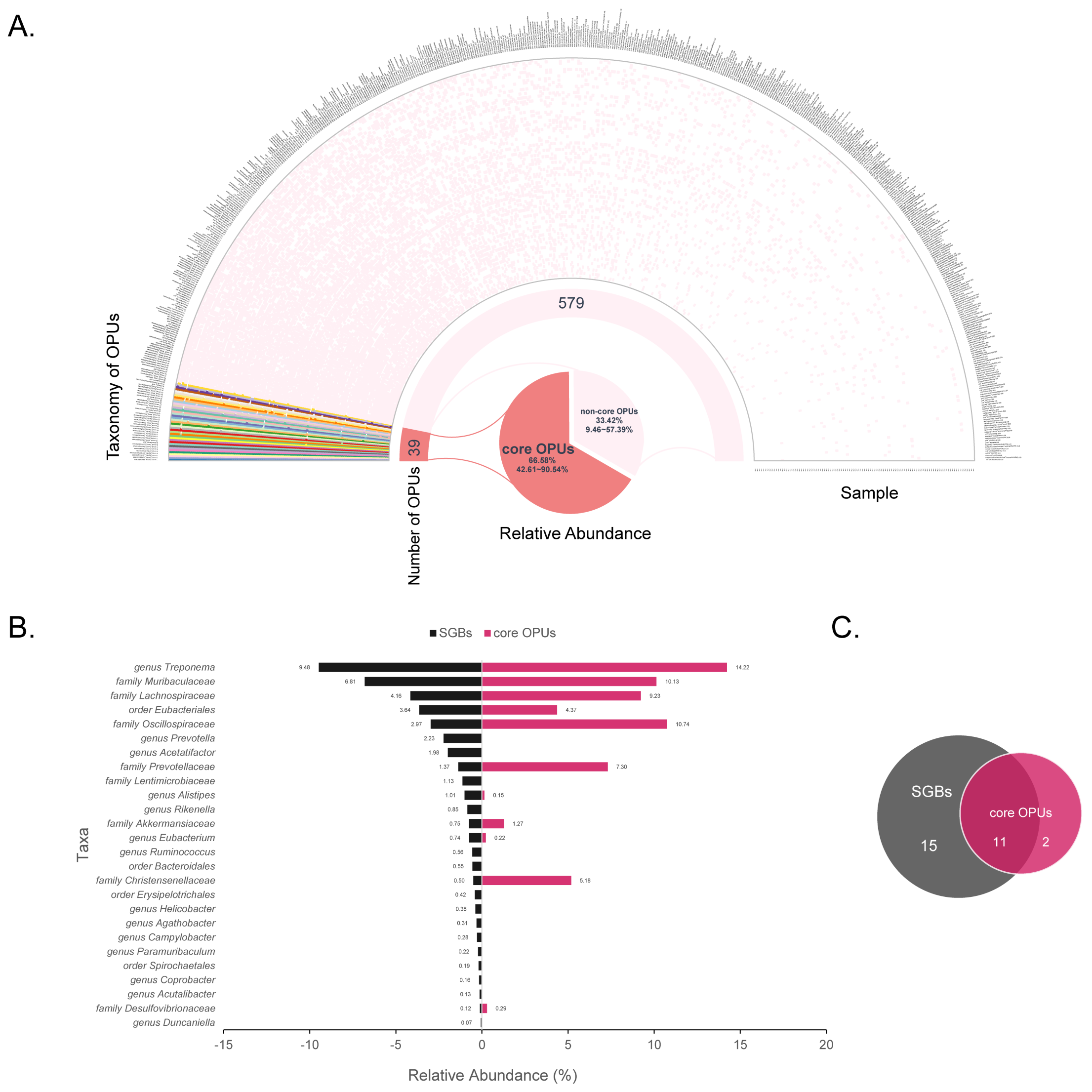

Figure S5. The core microbiota of intestinal content of Plateau Pika

(A) Heatmap and pie chart (relative abundance) of pika intestinal core OPUs distribution generated by 16s rRNA sequencing; Heatmap: each column represents an OPU, and each circle represents one sample, a different color represents each core OPU, pale pink represents the non-core OPUs; Pie chart: red represent the core OPUs and pale pink represents the non-core OPUs.

(B) Comparison of relative abundance between SGBs and core OPUs in the same samples;

(C) Venn diagram exhibit the consistency of SGBs and core OPUs.

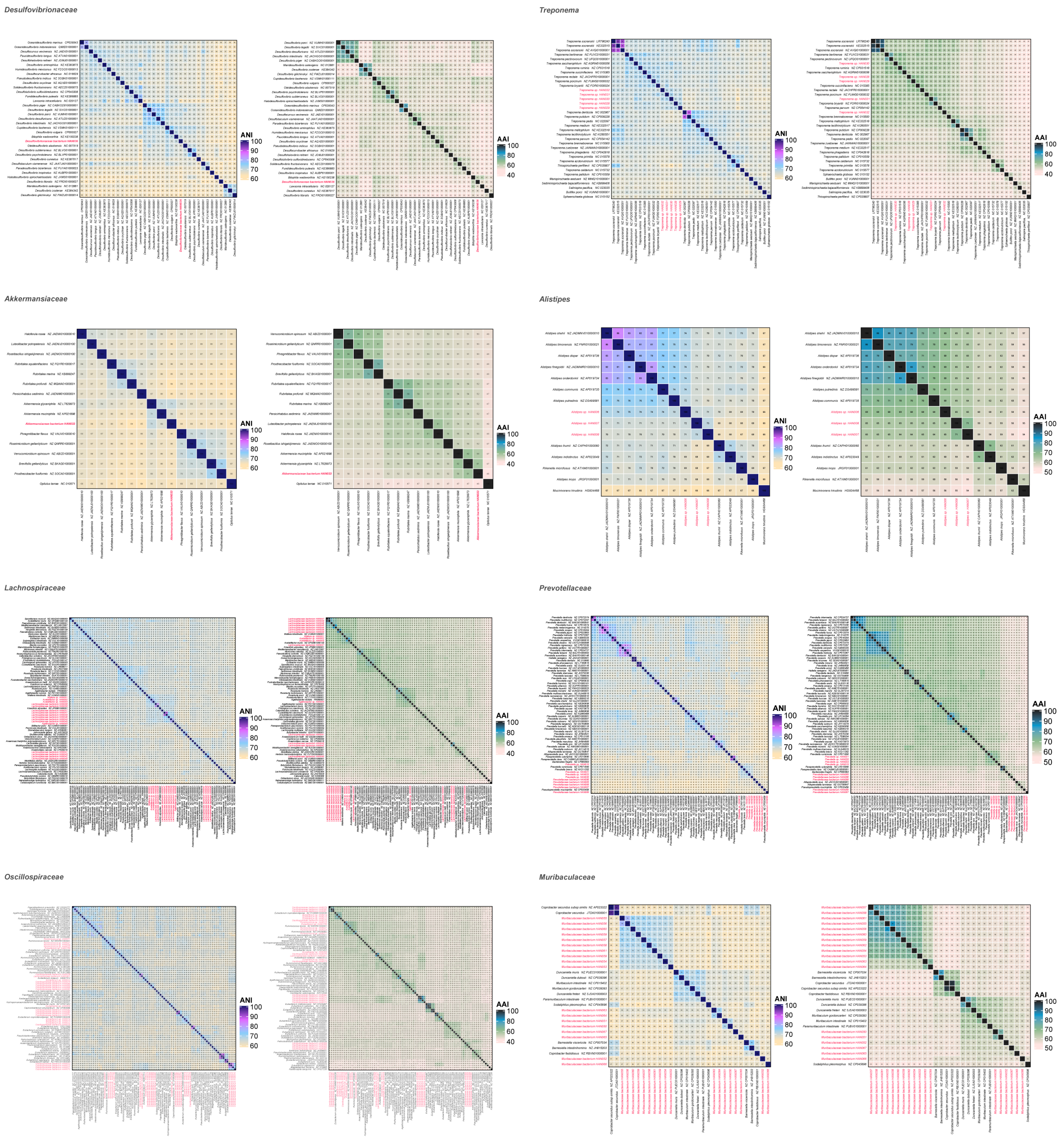

Figure S6. The AAI and ANI value of SGBs

The AAI value of SGBs compared with known species was calculated using compareM; The ANI value of SGBs compared with known species was calculated using orthoANI.

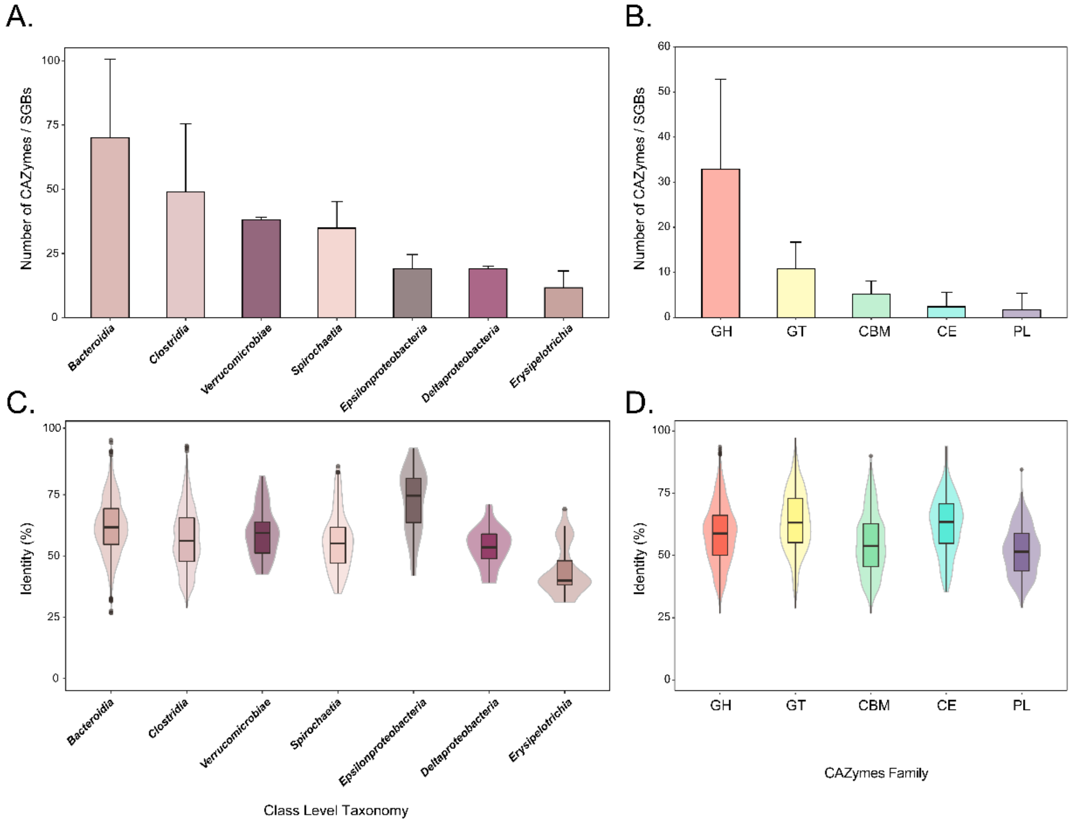

Figure S7. Profile of CAZymes detected in the SGBs

(A) Number of CAZymes detected per SGB under Class-level taxonomy;

(B) Number of CAZymes detected per SGB under different CAZymes families;

(C) Identities between the CAZyme sequences of SGBs from the different Class-level taxonomy and sequences in the CAZy database;

(D) Identities between the CAZyme sequences of SGBs from the different CAZymes families and sequences in the CAZy database.

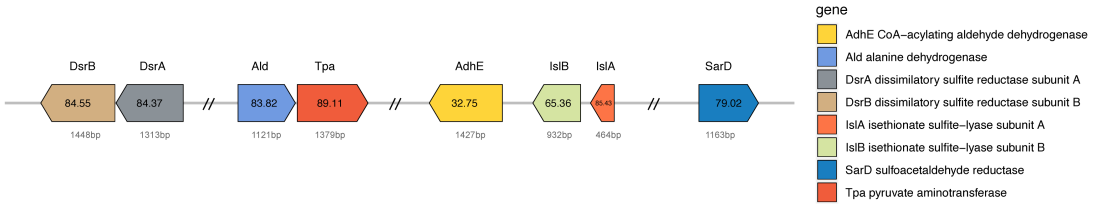

Figure S8. Taurine desulfonation pathway in SGBs

The gene clusters for metabolism of taurine identified in SGBs. Taurine transport and conversion to isethionate (*ald*, *tpa*, and *sarD*). Isethionate desulfonation and the conversion of acetaldehyde to acetyl-CoA (*adhE*, *islA*, and *islB*). Catalyzing the reduction of sulfite to form hydrogen sulfide (*dsrA* and *dsrB*)
